# Reassimilation of Photorespiratory Ammonium in *Lotus japonicus* Plants Deficient in Plastidic Glutamine Synthetase

**DOI:** 10.1371/journal.pone.0130438

**Published:** 2015-06-19

**Authors:** Carmen M. Pérez-Delgado, Margarita García-Calderón, Antonio J. Márquez, Marco Betti

**Affiliations:** Departamento de Bioquímica Vegetal y Biología Molecular, Facultad de Química, Universidad de Sevilla, Calle Profesor García González, Sevilla, Spain; Universidade Federal de Vicosa, BRAZIL

## Abstract

It is well established that the plastidic isoform of glutamine synthetase (GS_2_) is the enzyme in charge of photorespiratory ammonium reassimilation in plants. The metabolic events associated to photorespiratory NH_4_^+^ accumulation were analyzed in a *Lotus japonicus* photorespiratory mutant lacking GS_2_. The mutant plants accumulated high levels of NH_4_^+^ when photorespiration was active, followed by a sudden drop in the levels of this compound. In this paper it was examined the possible existence of enzymatic pathways alternative to GS_2_ that could account for this decline in the photorespiratory ammonium. Induction of genes encoding for cytosolic glutamine synthetase (GS_1_), glutamate dehydrogenase (GDH) and asparagine synthetase (ASN) was observed in the mutant in correspondence with the diminishment of NH_4_^+^. Measurements of gene expression, polypeptide levels, enzyme activity and metabolite levels were carried out in leaf samples from WT and mutant plants after different periods of time under active photorespiratory conditions. In the case of asparagine synthetase it was not possible to determine enzyme activity and polypeptide content; however, an increased asparagine content in parallel with the induction of *ASN* gene expression was detected in the mutant plants. This increase in asparagine levels took place concomitantly with an increase in glutamine due to the induction of cytosolic GS_1_ in the mutant, thus revealing a major role of cytosolic GS_1_ in the reassimilation and detoxification of photorespiratory NH_4_^+^ when the plastidic GS_2_ isoform is lacking. Moreover, a diminishment in glutamate levels was observed, that may be explained by the induction of NAD(H)-dependent GDH activity.

## Introduction

Nitrogen nutrition is one of the most crucial factors for plant productivity [1]. Nitrate is the main form of nitrogen used by many crops, but most plants are also able to use ammonium, amino acids and urea [2]. Symbiotic N_2_ fixation in the root nodule of legumes also provides important amount of reduced nitrogen to crops without the need of extensive fertilization [3]. NO_3_^-^ uptake by the roots is mediated by different families of transporters that are also involved in the sensing of this molecule [4]. Once inside the cell, NO_3_^-^ is first reduced to NO_2_^-^ in the cytoplasm by the action of nitrate reductase (NR; EC 1.7.1.1/2) and then further reduced to NH_4_^+^ by the action of nitrite reductase in the plastids (NiR; EC 1.7.7.1) [5]. The NH_4_^+^ that has been taken up directly from the soil or produced by the reduction of NO_3_^-^, atmospheric N_2_ in root nodules or urea hydrolysis, is then assimilated by the action of the glutamine synthetase (GS, EC 6.3.1.2)/glutamate synthase (GOGAT, EC 1.4.7.1 and 1.4.1.14) cycle. In addition to primary nitrogen assimilation, other pathways are able to produce NH_4_^+^ like amino acid catabolism, phenylpropanoid biosynthesis, asparagine breakdown and photorespiration in C3 plants [6]. Photorespiration is probably the most important of these pathways due to the very high flux and the different interconnections with other metabolic routes [7]. The large amounts of ammonium generated by the photorespiratory cycle are produced in the mitochondria as a result of the conversion of two molecules of glycine into one molecule of serine by the action of glycine decarboxylase (EC 2.1.2.10)/serine hydroxymethyl transferase multienzymatic complex (EC 2.1.2.1). NH_4_^+^ is transported to the chloroplast where it is reassimilated by the plastidic GS/GOGAT cycle.

Plants have two types of GS isoenzymes: the cytosolic isoform (or GS_1_), that is generally encoded by a small gene family composed of 3–5 members, and the plastidic GS isoform (or GS_2_), that is generally encoded by a single gene, with the exception of *Medicago truncatula* and poplar where two genes encoding for GS_2_ have been described [8–10]. While GS_1_ is the main enzyme involved in primary ammonium assimilation and recycling of the NH_4_^+^ generated during senescence and phenylpropanoid biosynthesis [11, 12], GS_2_ is mainly involved in the reassimilation of the ammonium generated during photorespiration. The key role played by GS_2_ in the reassimilation of photorespiratory ammonium was first demonstrated by the isolation of photorespiratory barley mutant that specifically lacked of this GS isoform [13, 14]. These mutants were conditionally lethal, since they grew well under a CO_2_-enriched atmosphere (>0.2% CO_2_), where photorespiration is suppressed, but showed stress symptoms and accumulated ammonium when transferred to normal CO_2_ conditions (about 0.04% CO_2_). Later on, oilseed rape plants with 50–75% lower GS_2_ activity were obtained using antisense technology [15]. However, these plants had low tissue levels of NH_4_^+^ since the residual GS_2_ activity was enough to efficiently reassimilate the ammonium produced during photorespiration. In our group, the first photorespiratory mutants deficient in GS_2_ from legume plants were isolated in the model legume *Lotus japonicus* [16]. These mutants lack completely of plastidic GS_2_ enzyme activity but have normal levels of cytosolic GS_1_ and were shown to be specifically affected in the gene encoding for plastidic GS_2_ [17] and accumulated high levels of ammonium when grown under active photorespiratory conditions.

A recent work from our group made use of a *L*. *japonicus* plastidic GS_2_ mutant called *Ljgln2-2* in order to study the transcriptomic and metabolic consequences of the lack of plastidic GS_2_ both under high and normal CO_2_ conditions [18]. A coordinate repression of the transcription of most photorespiratory genes was observed when the plants were shifted from high CO_2_ to normal CO_2_ conditions, as a way to avoid further accumulation of ammonium under active photorespiratory conditions. However, transcriptomic analysis indicated that important genes involved in N assimilation were modulated in the mutant under normal CO_2_ conditions, including genes encoding for cytosolic GS_1_ [18, 19]. This was part of a greater modulation of the transcriptome as well as of the metabolic profile of the mutant. These preliminary results indicated that other enzymes besides plastidic GS_2_ could be involved in the reassimilation of photorespiratory ammonium in the *Ljgln2-2* mutant.

Different enzymatic systems have been described in plants that may use ammonium [20]: 1) the GS/GOGAT cycle previously described. 2) Glutamate dehydrogenase (GDH, EC 1.4.1.2) deaminates glutamate to α-ketoglutarate (α-KG) and ammonium in order to replenish the TCA cycle of intermediates (NAD-dependent GDH activity) [21], but it has been also shown that the reverse reaction, also called aminating NADH-dependent GDH activity, may work in order to detoxify ammonium when the levels of this compounds are very high [22, 23]. In addition, a NADP(H)-GDH (EC 1.4.1.4) of unknown function also exists. 3) Asparagine synthetase (ASN, EC 6.3.5.4), whose main reaction is the incorporation of the amino group of glutamine into aspartate to yield asparagine [24, 25]. 4) Finally, carbamoylphosphate synthetase (CPS, EC 6.3.5.5) produces carbamoylphosphate (CP) from HCO_3_^-^, ATP and either NH_4_^+^ or glutamine [20].

The toxic effect that NH_4_^+^ produces in most plants is an interesting and still not fully understood phenomenon [26, 27]. For this reason, the study of the reactions involved in the assimilation/detoxification of both the external NH_4_^+^ obtained from the soil as well as the endogenous NH_4_^+^ produced by routes as the photorespiratory cycle is a topic of particular interest [19]. In this paper we studied the metabolic reactions that may be involved in NH_4_^+^ detoxification in the *Ljgln2-2* photorespiratory mutant. For this purpose, we carried out measurements of the transcript levels for the different sets of genes encoding for possible NH_4_^+^ detoxification enzymes in *L*. *japonicus* plants followed by determination of the corresponding polypeptide content and enzyme activities, as well as of the relative levels of the metabolites involved in the corresponding reactions at different time points. The results obtained indicate that, in the absence of plastidic GS_2_, different enzymes are induced in response to photorespiratory ammonium accumulation and may constitute an alternative way for photorespiratory ammonium reassimilation and detoxification in this plant.

## Materials and Methods

### Plant material and growth

*Lotus japonicus* (Regel) Larsen cv. Gifu was initially obtained from Professor Jens Stougaard (Aarhus University, Denmark) and then self-propagated at the University of Seville. WT and *Ljgln2-2* mutant plants were grown as described by Pérez-Delgado et al. [18]. For *Ljgln2-2*, the mutant progeny of two consecutive backcrosses into the WT background was used in this work. WT and mutant seeds were scarified and surface-sterilized, then germinated in 1% (w/v) agar in Petri dishes and transferred to pots using vermiculite as solid support. Five seedlings were planted in each pot and grown during 35 d in a growth chamber under 16 h: 8 h day: night, 20: 18°C, with a photosynthetic photon flux density of 250 μmol m^-2^ s^-1^ and a constant humidity of 70%. CO_2_ was automatically injected to a final concentration of 0.7% (v/v) (high CO_2_) to allow for normal growth of the *Ljgln2-2* mutant in a photorespiration-suppressed atmosphere. Plants were watered with “Hornum” nutrient solution, containing 5 mM NH_4_NO_3_ and 3 mM KNO_3_ [28]. After 35 days of growth under high CO_2_ atmosphere the plants had an average number of 7 trefoils. At this time, total leaf tissue was harvested for each plant genotype, constituting the time zero point (photorespiration suppressed conditions). All the leaf samples used in this work were harvested 4 hours after the beginning of the light period. Plants were then transferred to normal CO_2_ conditions (0.04% CO_2_ v/v) and each plant genotype was sampled at different time points (S1 Fig). Visible symptoms of the air sensitivity phenotype of the *Ljgln2-2* mutant plants started at the lower (older) leaves of the plants and progressed up to the younger leaves only after long periods of incubation under normal CO_2_ conditions that were not considered in this work. In any case, the 2–3 lower trefoils from the plants were always discarded to avoid any effect of senescence of the older leaves in our samples. Therefore, only the healthy leaves from the plants were harvested and pooled, and the corresponding tissue was flash-frozen in liquid N_2_, grinded with a pestle in a mortar that was pre-cooled with liquid N_2_ and the powder was stored at -80°C until use. Three independent biological replicates were harvested for each genotype and time point. A biological replicate consisted of tissue pooled from five plants grown in the same pot.

### RNA extraction, cDNA synthesis and qRT-PCR

RNA extraction was carried out using the hot borate method as described by [29]. cDNA synthesis was carried out with the Superscript III reverse transcriptase (Invitrogen) exactly as described by [18]. qRT-PCR was carried out in a LightCycler 480 thermal cycler (Roche) using the SensiFAST SYBR No-ROX Kit (Bioline) also using the same conditions described by [18]. Expression levels were quantified by determining the C_T_ cycle of each reaction with the LightCycler 480 software version 1.5.0. Expression data were standardized to the geometric mean of four housekeeping genes. The housekeeping genes were *LjGPI-*anchored protein (probeset: chr3.CM0047.42), *LjPp2A* (probeset: chr2.CM0310.22), *LjUbc10* (probeset: chr1.TM0487.4) and *LjUbq4* (probeset: chr5.CM0956.27); these genes were selected amongst the most stably expressed genes in plants [30]. A list of all the primers used for qRT-PCR is provided in the online version of this article (S1 Table).

### Preparation of crude extracts

Frozen leaf tissue was extracted with a pellet homogeniser in 5 ml/g fresh weight of extraction buffer. The homogenate was centrifuged for 15 min at 15,000 *g* and 4°C and the supernatant constituted the crude extract. The extraction buffer for GS activity assay and immunoblot was prepared according to [16]. For GDH activity and immunoblot the extraction buffer was 100 mM Tris·HCl pH 8 supplemented with 1% of polyvinylpolypyrrolidone. The extraction buffer for CPS activity assay was prepared according to [31]. Crude extracts for the assay of ASN activity were prepared in TrisˑHCl 50 mM pH 6.0.

### Western Blot

Western blots from denaturing PAGE were performed using the ECL Western blotting system (GE Healthcare) according to the manufacturer instructions. SDS-PAGE was performed according to the method of [32] using a Mini-Protean Tetra Cell system (Bio-Rad). An acrylamide concentration of 12%, 10% and 12.5% (w/v) was used in the SDS-PAGE running gels for immunodetection of GS, GDH and ASN respectively. Stacking gels were always prepared at 4% (w/v) acrylamide. 10 μg of total protein from leaf crude extracts were loaded in each well of the gels. Protein concentration was quantified using the Bradford protein assay (Bio-Rad). The polypeptide molecular weights were estimated using the broad range molecular weight standards (Bio-Rad).

Anti-GS primary antibodies were raised in rabbit immunised with the recombinant homopolymeric α GS from *Phaseolus vulgaris* purified by metal-affinity chromatography [33] and used at 1:1,000 dilution. Anti-GDH primary antibodies (a gift from Prof. K.A. Roubelakis-Angelakis, University of Crete) were raised in rabbit using purified NADH-GDH from grapevine [34]. Anti-ASN antibodies (a gift from Prof. FM Cánovas and RA Cañas, University of Málaga) were raised in rabbit using purified ASN from Scots pine (*Pynus sylvestris L*.) as described by [35]. Anti-rabbit secondary antibodies labelled with peroxidase were obtained from GE Healthcare.

Detection of peroxidase signal in the membranes was carried out with a quimioluminescence detection system (Fujifilm LAS 3000 mini, Fujifilm). Relative quantification of the polypeptide levels was carried out for three different biological replicates for each time point and genotype. Densitometry measurements were performed with the ImageJ software (http://rsb.info.nih.gov/ij/).

### Enzyme activity assays

Biosynthetic GS enzyme activity was determined by measuring ATP hydrolysis as described by [36]. GDH aminating and deaminating activities were measured as described by [37], except that 0.1 mM of NADH or NADPH was used in the aminating reaction. CPS enzyme activity was determined according to [38]. Assays for ASN enzyme activity were carried out according to [39].

### Metabolite profiling analysis

Metabolite profiling analysis was carried out using the dataset generated by Pérez-Delgado et al. [18]. Statistical differences between metabolite levels were assessed with two-way ANOVA at P < 0.001 using the Multiexperiment Viewer version 3.1 [40] and the factors “genotype” and “time under normal CO_2_”. Only metabolites that showed significant differences according to this analysis were considered. The relative metabolite levels presented in this paper are the direct normalized responses of metabolite pool measures, that is, mass detector signals in arbitrary units normalized to internal standard and sample fresh weigh.

Ammonia determination was carried out according to the method of [41] with some modifications as described by [16]. CP content in crude extracts was determined with the same colorimetric method used for CPS activity but using undialyzed crude extracts.

### Statistical analysis

All differences described in the text among WT and mutant plants and/or different time periods were confirmed to be significantly distinct using Student’s t test (p < 0.05)

## Results

### Ammonium accumulation and induction of cytosolic GS_1_ in the *Ljgln2-2* mutant

The levels of ammonium and of several other metabolites involved in NH_4_^+^ assimilation and metabolism like Glu, Gln, α-KG, Asp, Asn, Gly, Ser, Lys and CP were measured in leaves of WT and *Ljgln2-2* mutant plants at different time points of the transfer from high CO_2_ (suppressed photorespiration) to normal CO_2_ (active photorespiration) (Table 1). The levels of glycine and serine were indicative of the existence of a manifest photorespiratory carbon flow both in WT and mutant plants. As it would be expected, an important increase in ammonium levels could be observed in the mutant plants compared to the WT, which was due to the lack of plastidic GS_2_ in the mutants. The presence of normal levels of plastidic GS_2_ in the WT plants made them able to efficiently reassimilate the photorespiratory ammonium in contrast to the *Ljgln2-2* mutant plants. However, interestingly, a gradual decline of these ammonium levels was detected in the mutants after the maximum reached at day 3. qRT-PCR analysis showed a coordinate repression of the genes involved in photorespiration in the *Ljgln2-2* mutant after the transfer to active photorespiratory conditions [18], suggesting that the mutant may down regulate the photorespiratory pathway in order to avoid further accumulation of ammonium. In addition, the sudden drop in ammonium levels observed in *Ljgln2-2* mutant plants after three days of the transfer to normal air was suggestive of the induction of alternative routes for the reassimilation of photorespiratory ammonium that was accumulated in this genotype. The analysis of these routes was further explored.

**Table 1:** Metabolite levels in WT and mutant plants.

Days in normal CO_2_								
		0	2	3	4	6	8	10
NH4+								
	WT	**0.0 ± 0.0**	**0.4 ± 0.1**	**0.4 ± 0.1**	**2.6 ± 2.8**	**0.7 ± 0.1**	**0.3 ± 0.0**	**0.5 ± 0.2**
	*Ljgln2-2*	**3.0 ± 2.0**	**45.6 ± 0.5**	**64.4 ± 1.1**	**52.7 ± 1.0**	**30.9 ± 8.2**	**21.7 ± 2.3**	**26.0 ± 3.4**
Glu								
	WT	12.9 ±3.1	**11.1 ± 3.0**	**9.5 ± 0.5**	**12.0 ± 2.0**	**10.5 ± 0.2**	**13.6 ± 2.8**	**11.7 ± 1.5**
	*Ljgln2-2*	9.7 ± 1.1	**4.8 ± 0.5**	**4.4 ± 0.7**	**5.9 ± 0.8**	**7.4 ± 0.5**	**7.3 ± 3.1**	**5.5 ± 0.9**
Gln								
	WT	6.8 ± 2.7	3.1 ± 2.4	**4.2 ± 0.6**	**1.9 ± 0.6**	**2.4 ± 0.0**	**3.8 ± 1.3**	**2.3 ± 0.6**
	*Ljgln2-2*	3.7 ± 1.4	6.8 ± 2.5	**8.0 ± 1.3**	**13.0 ± 2.8**	**20.1 ± 8.7**	**23.4 ± 5.7**	**16.5 ± 1.6**
α-KG								
	WT	0.9 ± 0.4	**0.7± 0.4**	**0.8 ± 0.7**	**0.4 ± 0.1**	**0.2 ± 0.0**	**0.5 ± 0.2**	**0.4 ± 0.1**
	*Ljgln2-2*	0.5 ± 0.1	**13.9 ± 2.7**	**21.4 ± 5.7**	**9.1 ± 2.7**	**3.3 ± 0.7**	**4.4 ± 1.5**	**2.5 ± 0.7**
Asp								
	WT	**14.6 ± 4.9**	**13.2 ± 3.1**	**10.1 ± 0.6**	**12.8 ± 0.7**	**16.3 ± 1.7**	**17.5 ± 3.8**	**12.9 ± 2.2**
	*Ljgln2-2*	**7.9 ± 0.7**	**4.7 ± 0.4**	**3.2 ± 0.1**	**4.6 ± 0.8**	**10.4 ± 4.7**	**7.8 ± 3.5**	**5.3 ± 0.4**
Asn								
	WT	14.2 ± 5.6	8.5 ± 3.2	10.8 ± 2.9	**6.3 ± 0.3**	**6.5 ± 0.1**	9.4 ± 1.9	**7.9 ± 1.4**
	*Ljgln2-2*	8.5 ± 1.2	5.7 ± 0.8	8.4 ± 0.2	**10.9 ± 0.7**	**12.1 ± 2.0**	13.6 ± 5.3	**12.1 ± 1.0**
Gly								
	WT	0.6 ± 0.1	0.7 ± 0.3	**0.6 ± 0.1**	**0.5 ± 0.1**	**0.4 ±0.1**	**0.7 ± 0.2**	**0.4 ± 0.2**
	*Ljgln2-2*	0.7 ± 0.1	0.9 ± 0.1	**1.7 ± 0.3**	**1.3 ± 0.2**	**1.3 ± 0.2**	**1.3 ± 0.2**	**0.8 ± 0.1**
Ser								
	WT	**7.9 ± 1.2**	**11.9 ± 2.2**	**10.2 ± 1.2**	**10.0 ± 0.7**	**8.3 ± 0.1**	13.8 ± 3.6	**9.5 ± 1.3**
	*Ljgln2-2*	**11.5 ± 2.2**	**16.7 ± 1.0**	**22.9 ± 3.8**	**15.0 ± 1.8**	**19.7 ± 6.0**	18.2 ± 4.8	**12.0 ± 0.9**
Lys								
	WT	2.3 ± 0.3	**1.8 ± 0.4**	**1.9 ± 0.6**	**1.5 ± 0.5**	**2.0 ± 0.7**	3.5 ± 2.6	**2.1 ± 0.7**
	*Ljgln2-2*	1.7 ± 0.5	**9.1 ± 2.0**	**5.9 ± 3.5**	**5.4 ± 0.5**	**5.3 ± 1.1**	5.1 ± 0.9	**3.9 ± 0.2**
CP *								
	WT	**0.15± 0.08**	**0.35 ± 0.11**	**0.28 ± 0.10**	**0.21 ± 0.13**	0.19 ± 0.15	0.17 ± 0.14	**0.18 ± 0.12**
	*Ljgln2-2*	**0.05± 0.02**	**0.07 ± 0.04**	**0.01 ± 0.01**	**0.01 ± 0.03**	0.05 ± 0.01	0.04 ± 0.02	**0.01 ± 0.01**

The levels of different metabolites were determined from leaves of WT and *Ljgln2-2* mutant plants grown in high CO_2_ conditions (0 days) and after different periods of time under normal CO_2_ conditions. Metabolite levels are expressed as relative units with the exception of NH_4_^+^ and CP that are expressed in μmol·g FW^-1^. Numbers in bold represent significant differences between WT and mutant genotypes at this time point according to Student’s t test (p<0.05).

*CP levels have two decimal digits since they were measured using a different technique compared to the other metabolites.

**Fig 1:**
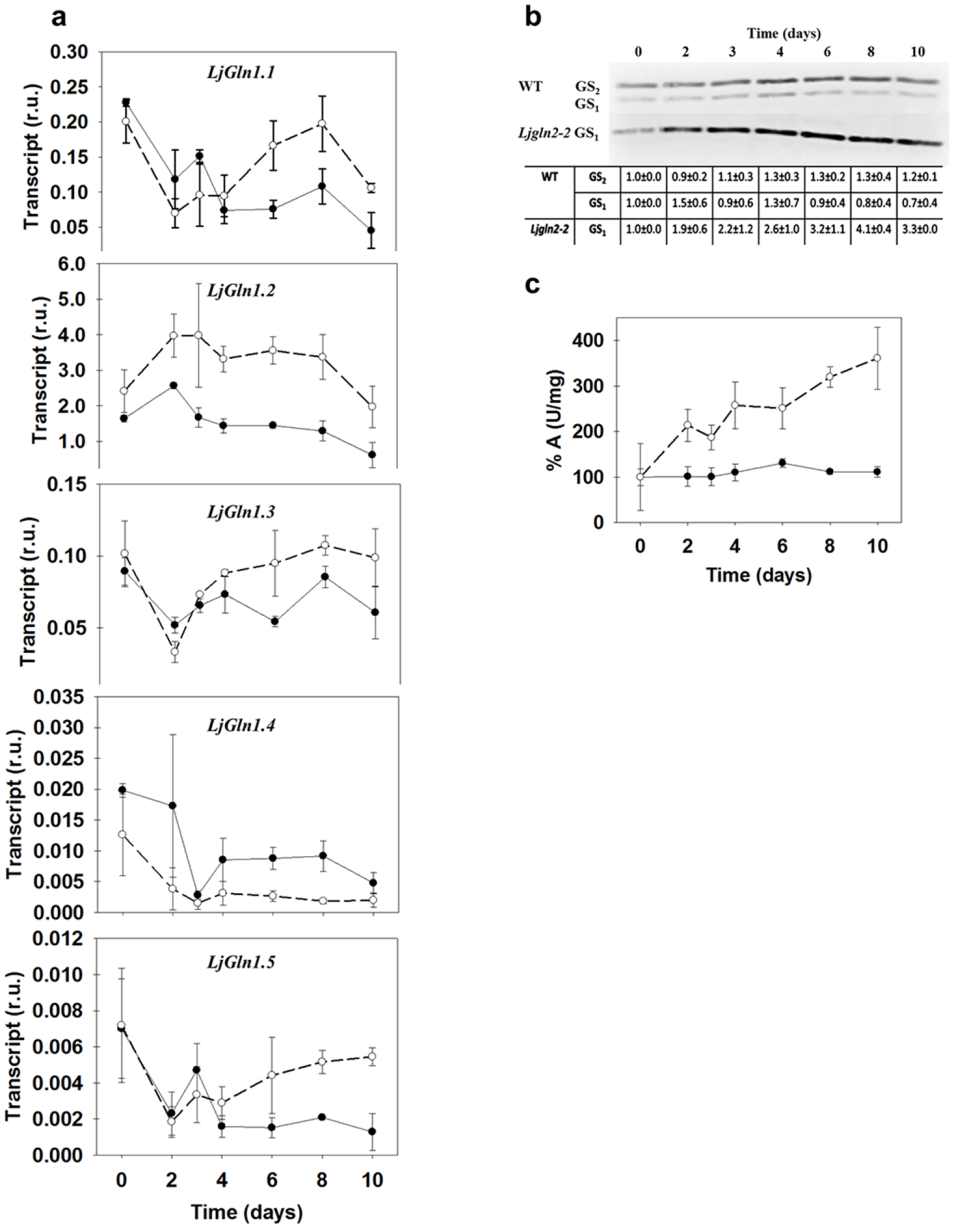
Changes in GS gene expression and activity in WT and *Ljgln2-2* mutant plants. **(a)** Expression of the genes for cytosolic GS_1_ in WT and mutant plants. Transcript levels were quantified by qRT-PCR in WT (black dots, solid line) and mutant (white dots, dashed line) leaves at the time points indicated. Relative transcript levels are reported for each genotype compared to the housekeeping genes. **(b)** Immunodetection of cytosolic GS_1_ and plastidic GS_2_ in leaf crude extracts from WT and mutant plants. 10 μg of total protein were loaded on each lane. The position of the molecular weight marker (kDa) is indicated at the right side of the blot. The table reports the densitometric quantification of the GS_1_ and GS_2_ bands relative to their values at time zero which were taken as 1. Data are the mean ± SD of three independent biological replicates. The blot shown in the Figure is only one of the different replicates analyzed. **(c)** The GS specific activity was determined in crude extracts from leaves of WT (black dots, solid line) and *Ljgln2-2* (white dots, dashed line). GS activity is shown as specific activity (U/mg of total protein). Data are the mean ± S.D. of three independent biological replicates.

The levels of expression of the different GS_1_ genes were determined both for WT and mutant plants at different time points under active photorespiratory conditions. In order to do that, all the sequences corresponding to GS_1_ were retrieved from the Kazusa database (www.kazusa.or.jp/e/). A total of five unique gene sequences encoding for cytosolic GS_1_ were found (see S1 Table for the accession numbers). Gene specific oligonucleotides were used in order to measure the mRNA levels in WT and mutant plants by qRT-PCR at the different time points considered. *LjGln1*.*2* was the most highly expressed GS_1_ gene in leaves and was induced almost two-fold in the mutant, with a maximum in transcript levels at day 3 followed by a slow decrease (Fig 1a). *LjGln1*.*1*, *LjGln1*.*3* and *LjGln1*.*5* genes showed similar expression profiles, with a sudden decrease in transcript levels in both genotypes, followed by a recovery in transcript levels at days 6–8 exclusively in the mutant. Finally, *LjGln1*.*4* was expressed at very low levels and was repressed in both genotypes after the shift to normal CO_2_ conditions. All these results indicated that the different genes encoding for cytosolic GS_1_ were modulated by the transfer to active photorespiratory conditions in both genotypes.

In order to determine if these changes in gene expression were paralleled by changes in polypeptide levels, a Western blot analysis was carried out using anti-GS antibodies that recognize both cytosolic and plastidic GS. Cytosolic GS_1_ polypeptide levels were increased in the mutant during the time-course of the experiment. The maximum levels of GS_1_ polypeptide were observed after eight days under normal CO_2_ conditions, where the polypeptide levels were about four times higher than in the WT (Fig 1b).

Total GS enzyme activity was also increased exclusively in *Ljgln2-2* in good agreement with GS_1_ polypeptide levels, indicating that the increased amount of GS_1_ was represented by active enzyme (Fig 1c). Moreover, glutamine levels were also increased in the mutant in parallel with GS_1_ polypeptide levels and total GS activity (Table 1). These data suggest a role for cytosolic GS_1_ as an alternative enzyme for photorespiratory NH_4_^+^ reassimilation and detoxification in GS_2_-deficient plants. In contrast, a drop in glutamine levels was observed in WT plants after the transfer to active photorespiratory conditions (Table 1).

### Induction of different GDH activities in the *Ljgln2-2* mutant

**Fig 2:**
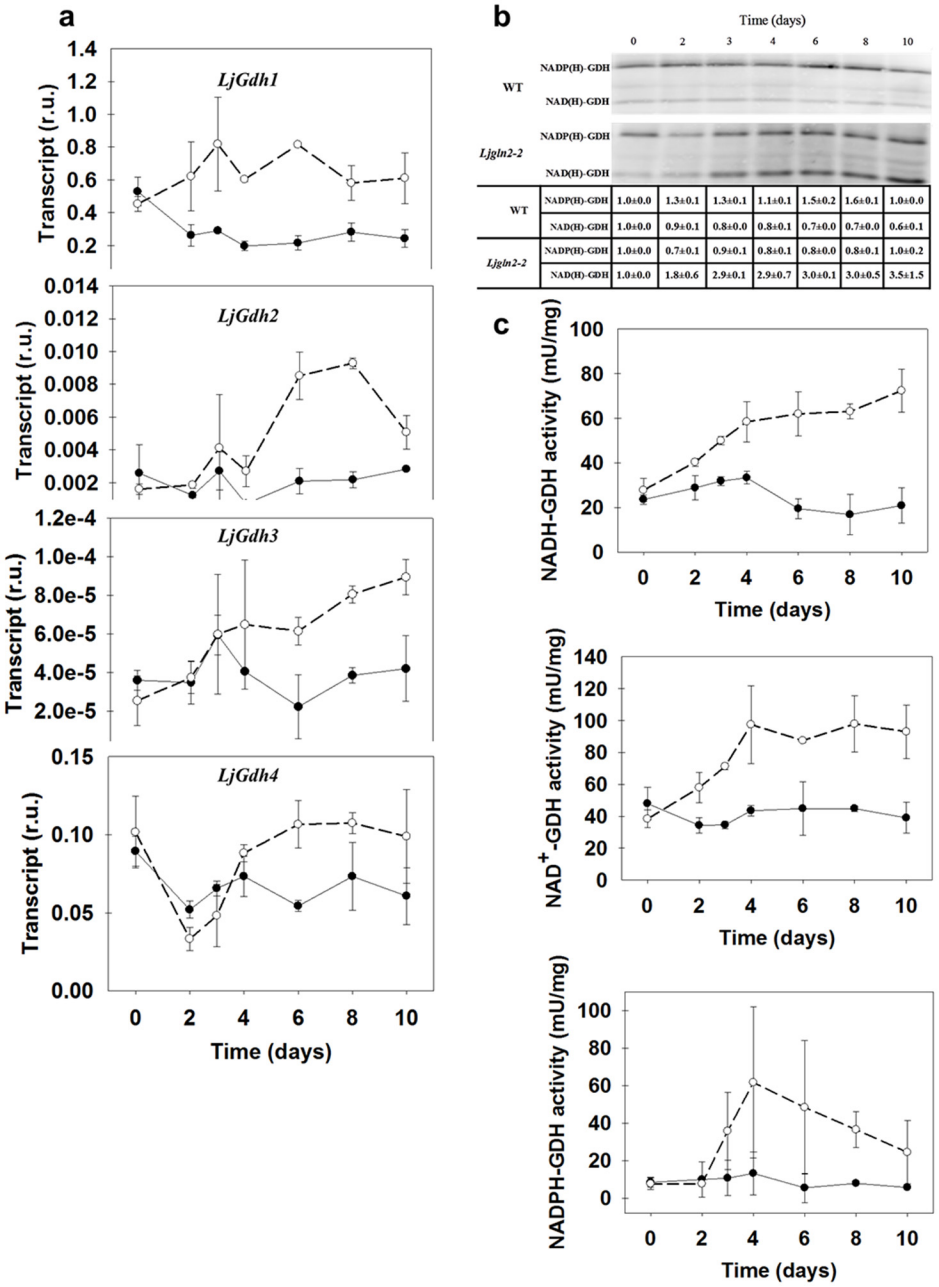
Changes in GDH gene expression and activity in WT and *Ljgln2-2* mutant plants. **(a)** Expression of the genes for NAD(H)-GDH (*LjGdh1*, *LjGdh2* and *LjGdh3*) and NADP(H)-GDH (*LjGdh4*) in WT and mutant plants. Transcript levels were quantified by qRT-PCR in WT (black dots, solid line) and mutant (white dots, dashed line) leaves at the time points indicated. Relative transcript levels are reported for each genotype compared to the housekeeping genes. **(b)** Immunodetection of NAD(H)-GDH and NADP(H)-GDH in leaf crude extracts from WT and mutant plants. 10 μg of total protein were loaded on each lane. The position of the molecular weight markers (kDa) is indicated at the right side of the blot. The table reports the densitometric quantification of the NAD(H)-GDH and NADP(H)-GDH bands relative to their values at time zero which were taken as 1. Data are the mean ± SD of three independent biological replicates. The blot shown in the Figure is only one of the different replicates analyzed. **(c)** GDH specific activity was determined in crude extracts from leaves of WT (black dots, solid line) and *Ljgln2-2* (white dots, dashed line). GDH activity was measured in both directions using either NAD^+^, NADH, NADP^+^ or NADPH as co-factors. NADP^+^-dependent GDH activity was undetectable in both genotypes and is not shown here. GDH activity is shown as specific activity (mU/mg of total protein). Data are the mean ± S.D. of three independent biological replicates.

Three different gene sequences encoding for NAD(H)-GDH (*LjGdh1*, *LjGdh2* and *LjGdh3*) as well as a fourth gene encoding for a putative NADP(H)-dependent GDH enzyme (*LjGdh4*) were found in the *L*. *japonicus* genome. The experimental molecular weights of the corresponding polypeptides depicted from the gel were of about 45 kDa for the NAD(H)-GDH isoforms and of about 71 kDa for the NADP(H)-GDH one, that are in good agreement with the theoretical molecular weights of about 44.7 kDa and 70.7 kDa calculated for the NAD(H)-dependent and NADP(H)-dependent isoforms respectively (S2 Fig). Two of the three genes encoding for NAD(H)-GDH were induced in the mutant and were not significantly modulated in the WT after four days under normal CO_2_ conditions (*LjGdh2* and *LjGdh3*, Fig 2a). On the other hand, the most highly expressed GDH gene in leaves (*LjGdh1*) was significantly induced in the mutant after the transfer to normal CO_2_ conditions and it was repressed about three-fold in the WT genotype. *LjGdh4* transcript levels showed in both genotypes a sudden drop after 2 days under active photorespiration followed by a clear increase taking place in the mutant plants, with a trend that was almost opposite to NH_4_^+^ levels.

Two main immunoreactive bands were detected by Western blot analysis using anti NAD(H)-GDH antibodies from grapevine: one compatible with the size expected for the NAD(H)-GDH polypeptides (about 45 kDa for all the three possible isoforms) and another one with a higher molecular weight that was compatible with the size of the putative NADP(H)-GDH polypeptide (about 71 kDa). Cross-reactivity between NAD(H)-GDH antibodies and NADPH-GDH may be expected. Sequence alignment of the *L*. *japonicus* NADP(H)-GDH protein (*LjGDH4*) with the other three NAD(H)-GDH protein sequences showed the presence of two highly conserved domains between the two types of GDH: a dehydrogenase dimerization domain (pfam02812), between the amino acid residues 240 and 360 of LjGDH4, and the GDH NAD(P)-binding domain, from the amino acid residue 379 until the end of the LjGDH4 sequence (S2 Fig). These highly conserved regions between NAD(H)-GDH and NAD(P)H-GDH may account for the cross-reactivity observed for the anti NAD(H)-GDH antibody. Quantification of the immunoblots indicated an increase of up to three times in NAD(H)-GDH polypeptide levels in the mutant, while in the WT they were almost unchanged (Fig 2b). On the other hand, NADP(H)-GDH polypeptide levels were higher in the WT while in the mutant plants they showed an initial decrease followed by a substantial recovery along the time in parallel with the levels found for the *LjGdh4* transcript.

The levels of NAD(H)-GDH enzyme activity were determined both in the aminating and deaminating directions. Both assays indicated an increase in activities of about 3–4 times exclusively in the mutant, in very good agreement with the polypeptide levels. On the other hand, the NADP(H)-GDH enzyme activity was also induced in the mutant, although with more variation between biological replicates compared to the NADH-GDH and NAD^+^-GDH activities (Fig 2c). In this case, only the aminating NADPH-dependent GDH enzyme activity was detectable, showing an increase in parallel with the increase in transcript levels observed for the corresponding *LjGdh4* gene. This increase in NADPH-dependent GDH activity took place concomitantly with the decrease in ammonium levels observed in *Ljgln2-2* mutants after three days of the transfer to normal CO_2_ (Table 1). The NADP^+^-dependent deaminating activity was undetectable in WT and mutant extracts (not shown). These results are very interesting considering that NADP(H)-GDH is still a poorly characterized enzyme in plants.

**Fig 3:**
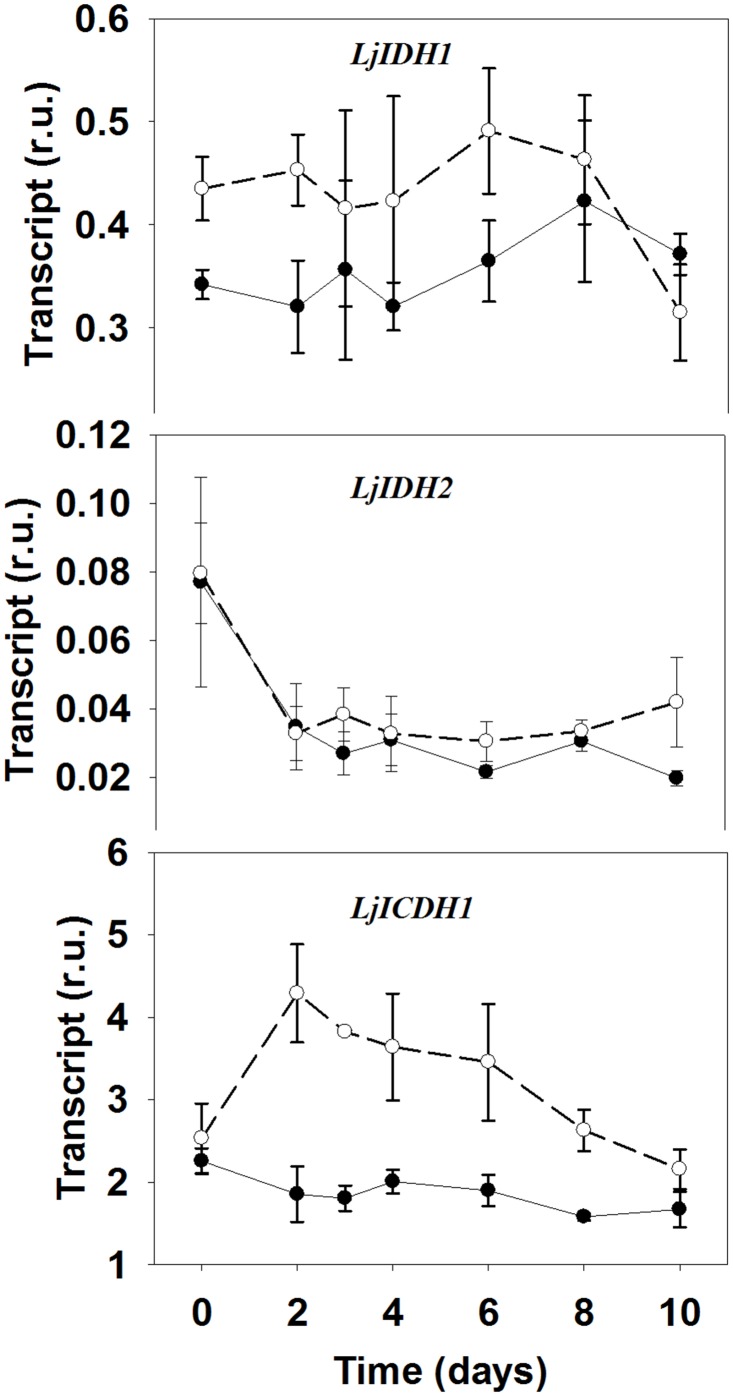
Expression levels of genes encoding for different IDH and ICDH isoforms in WT and *Ljgln2-2* mutant plants. Transcript levels were quantified by qRT-PCR in WT (black dots, solid line) and mutant (white dots, dashed line) leaves at the time points indicated. Relative transcript levels are reported for each genotype compared to the housekeeping genes. Data are the mean ± S.D. of three independent biological replicates.

Analysis of the metabolite levels corresponding to the substrates and products of GDH reaction showed that the levels of α-KG were increased in the mutant, with a trend over time very similar to the ammonium one (Table 1). On the other hand, glutamate leaf content decreased about two-fold in the mutant genotype after the shift to active photorespiratory conditions. These data suggested that GDH activity may contribute to provide the carbon skeletons necessary for the reassimilation of photorespiratory NH_4_^+^ by means of Glu deamination. However, α-KG can be also produced by other metabolic reactions, most importantly by the oxidative decarboxylation of isocitrate by the action of isocitrate dehydrogenase (called IDH or ICDH depending on whether the cofactor is NADH or NADPH, [42]). Moreover, Lys catabolism has been also proved able to evolve α-KG as a terminal product [43]. The latter option seemed improbable since Lys accumulated about 3–5 times in the mutant under active photorespiratory conditions (Table 1). However, qRT-PCR analysis of transcript levels for different genes encoding for isocitrate dehydrogenase showed that *LjICDH1*, which encodes for a NADP-dependent ICDH predicted to be localized in the cytosol, was highly induced in the mutant (Fig 3). Interestingly, the transcript levels for *LjICDH1* were parallel to the α-KG metabolite ones, suggesting that this gene may also contribute to the increase in α-KG detected in the mutant. On the other hand, the most transcribed gene encoding for the NAD-dependent isocitrate dehydrogenase isoform, *LjIDH1*, did not show any clear regulation after the transfer to active photorespiratory conditions, while *LjIDH2*, the second gene encoding for IDH in *L*. *japonicus*, was repressed in both genotypes under the same conditions (Fig 3).

### Transcriptional modulation of ASN genes

Two genes were initially described as encoding for ASN in *L*. *japonicus* [44]: *LjAS1* and *LjAS2*. Here we will call these two genes *LjAsn1* and *LjAsn2* respectively according to the current nomenclature for asparagine synthetase genes. However, mining of the available database information led to the identification of a third *Asn* gene, that we will call *LjAsn3*.

**Fig 4:**
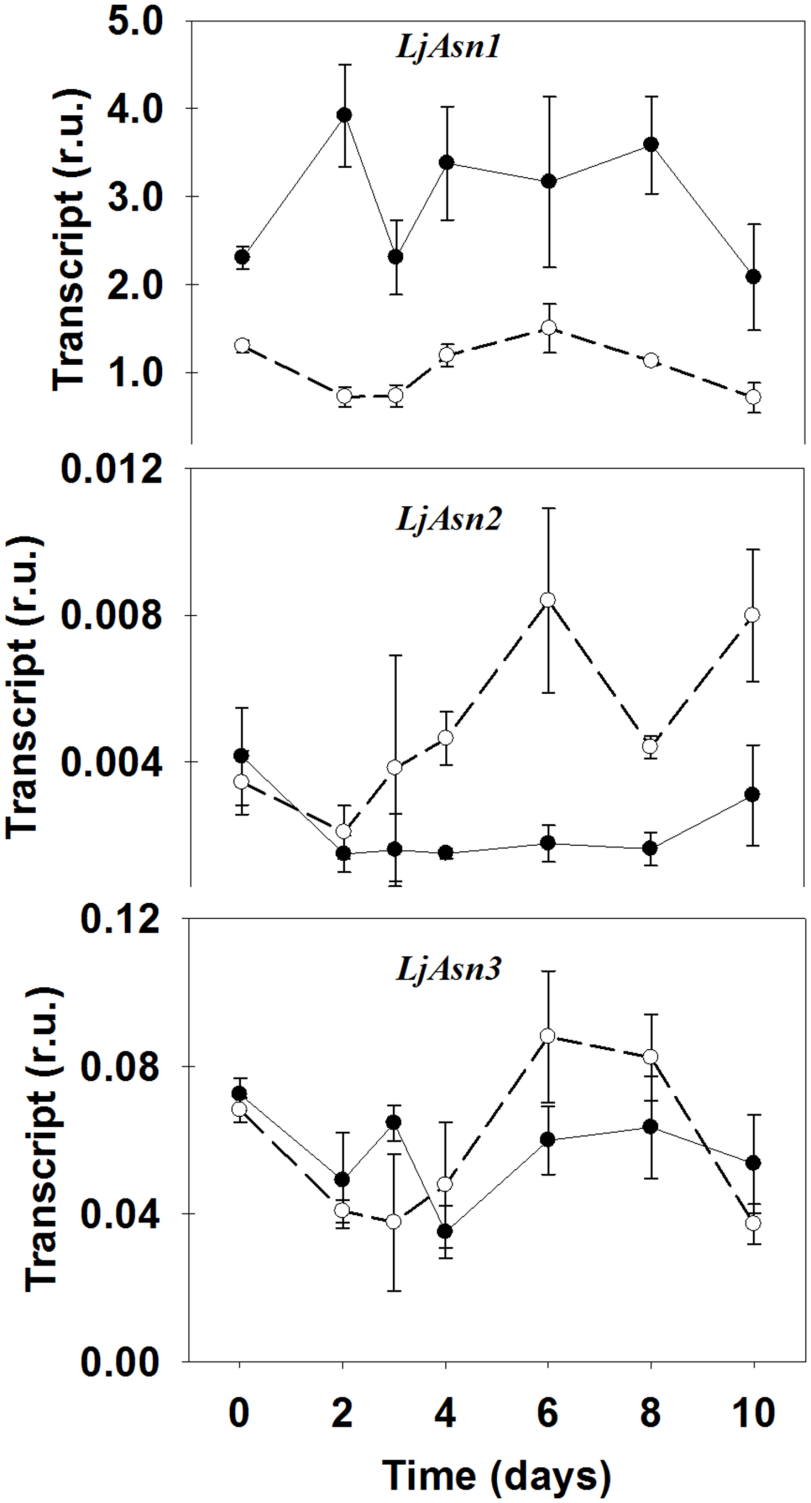
Expression levels of genes encoding for different ASN isoforms in WT and *Ljgln2-2* mutant plants. Transcript levels were quantified by qRT-PCR in WT (black dots, solid line) and mutant (white dots, dashed line) leaves at the time points indicated. Relative transcript levels are reported for each genotype compared to the housekeeping genes. Data are the mean ± S.D. of three independent biological replicates.

Information about the role of ASN in plants came mainly from measurements of gene expression and metabolite levels. This is because of the difficulty in determining ASN polypeptide levels and enzyme activity due to the rapid turnover of the enzyme, to the presence of asparaginase activity and of ASN inhibitors in the extracts [45]. This was also the case of *L*. *japonicus* leaves extracts since it was not possible to determine ASN enzyme activity and no immunoreactive bands were found using anti-ASN primary antibodies (results nor shown). However, qRT-PCR analysis showed a clear induction of the *LjAsn2* gene, the less expressed of the three ASN genes, exclusively in the mutant (Fig 4). *LjAsn1*, the most expressed one, did not show a clear trend over the time-course of the experiment but it was always more expressed in the WT than in the mutant, even at time zero (high CO_2_ conditions) where most genes showed similar expression levels in the two genotypes. No significant differences between WT and *Ljgln2-2* mutants were detected for the expression of the *LjAsn3* gene except for a minor increase observed in the mutants at days 6 and 8.

Interestingly, a significant accumulation of asparagine was observed in the *Ljgln2-2* mutant at four, six and ten days of permanence under active photorespiratory conditions (Table 1), suggesting that asparagine biosynthesis may be increased in the mutant plants in accordance with the data obtained on *LjAsn2* gene expression (Fig 4). On the other hand, aspartate levels were always lower in the mutant plants compared to the WT thus suggesting that aspartate may be used for the synthesis of asparagine and/or other uses in the mutant plants.

### CPS gene expression and CP content in WT and *Ljgln2-2*

**Fig 5:**
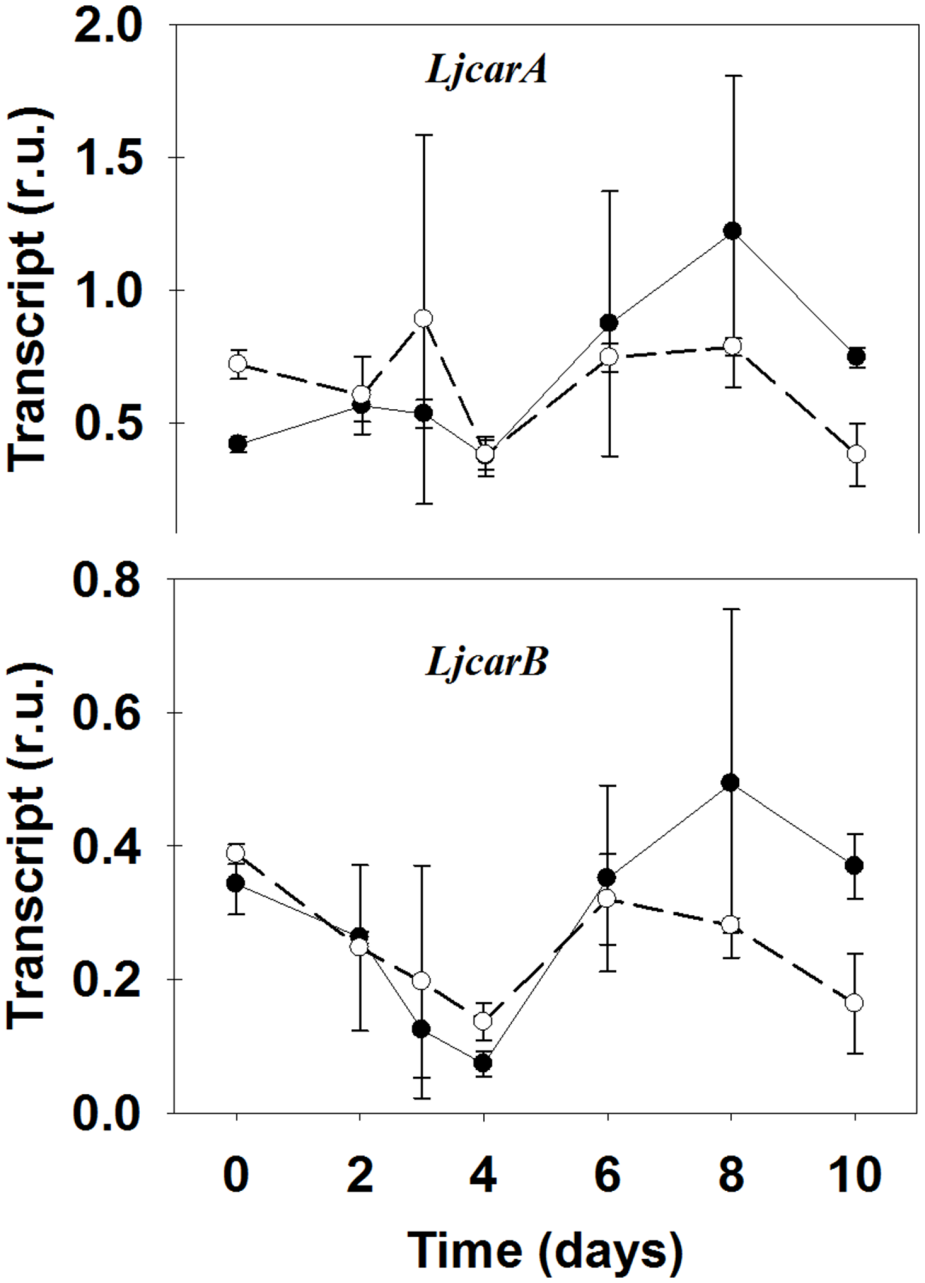
Expression levels of genes encoding for CPS in WT and *Ljgln2-2* mutant plants. Transcript levels for the genes encoding for the CPS small subunit (*LjCarA*) and for the large subunit (*LjCarB*) were quantified by qRT-PCR in WT (black dots, solid line) and mutant (white dots, dashed line) leaves at the time points indicated. Relative transcript levels are reported for each genotype compared to the housekeeping genes. Data are the mean ± S.D. of three independent biological replicates.

Plant CPS is similar to the bacterial enzyme and is formed by a large and a small subunit [46]. Most plants have two genes encoding for the small and large CPS subunits respectively, that in Arabidopsis are called *carA* and *carB* [20]. In *L*. *japonicus*, two homologous genes for CPS were found: *LjCarA* and *LjCarB*. The two genes showed similar levels of expression in *L*. *japonicus* leaves (Fig 5). The transfer to active photorespiratory conditions produced a repression of about three times in the expression of *LjCarB* gene after four days of active photorespiration followed by a recovery of transcript levels in both genotypes while no clear differences were obtained in the expression levels of *LjCarA* gene. No detectable levels of CPS activity were found in *L*. *japonicus* leaf extracts, either in WT or mutant plants. However, it was found that the levels of carbamoylphosphate were always much higher in the WT than in the *Ljgln2-2* mutant plants and, in the case of WT plants, CP increased more than two-fold with a peak at day 2 followed by a sudden decrease (Table 1).

## Discussion

*Ljgln2-2* mutant plants accumulated high levels of NH_4_^+^ under active photorespiratory conditions followed by a sudden decrease in the content of this compound. This was suggestive of a regulation of photorespiratory metabolism and/or of enzymes involved in secondary NH_4_^+^ assimilation in the *Ljgln2-2* mutants. Since the transcriptional regulation of photorespiratory and photosynthetic genes has been already described [18], the present paper was focused on the analysis of the different sets of enzymes that may be involved in ammonium reassimilation and detoxification of photorespiratory NH_4_^+^ and could account for the drop in this compound observed in the *Ljgln2-2* mutant.

While the high accumulation of NH_4_^+^ observed in *Ljgln2-2* clearly indicates that the enzyme involved in the re-assimilation of photorespiratory NH_4_^+^ is plastidic GS_2_, the induction of cytosolic GS_1_ activity, together with the increase in Gln levels, suggested that GS_1_ may be induced in order to contribute to the reassimilation and detoxification of this compound. This would be in agreement with previous reports that have shown that overexpression of cytosolic GS_1_ in leaf mesophyll cells of tobacco resulted in a reduction of free ammonium levels in leaves under active photorespiratory conditions [47]. In addition, particular isoforms of cytosolic GS_1_ such as GLN1.2 in *Arabidopsis* were suggested to be essential for ammonium detoxification [48]. Other recent data also indicate that GS_1_ could have an important role under ammonium nutrition [49, 50]. It has to be pointed out that cytosolic GS_1_, as well as ASN and GDH are induced in old senescing leaves in order to reassimilate the NH_4_^+^ generated by protein degradation [5]. However, we exclude the possibility that the changes observed in the present paper may be due to the onset of the senescence because young plants and healthy leaves were always analyzed at the different time periods examined and no proper symptoms of senescence were detected (no protein or nucleic acid degradation or other senescence parameters were observed at 10 days of transfer). In fact, most of the modulations in gene expression and enzyme activities observed in this paper started to take place at a very short time after the transfer from high CO_2_ to normal air conditions making it very unlikely that they could be attributed to senescence. The sudden modulation of gene expression and enzyme activities upon transfer from high CO_2_ to normal air conditions, rather support the idea that the events described in this paper represent an early response to the increased levels of photorespiratory NH_4_^+^. Moreover, the peak in NH_4_^+^ level and the subsequent drop were clearly observed before any symptoms of the air-sensitivity phenotype can be detected.

It has to be taken into consideration that the different products of the cytosolic GS_1_ gene family are generally located in different cells of the vascular tissue, while plastidic GS_2_ is normally located in mesophyll cells where photorespiratory metabolism occurs [11, 51]. If this is the case in *L*. *japonicus*, then some transport or diffusion mechanism between photosynthetic and vascular tissues should function in order to shift the accumulated NH_4_^+^ in the mutant to the tissues were cytosolic GS_1_ is present. However, it should be also taken into consideration that under certain stress conditions the presence of cytosolic GS_1_ in mesophyll cells has been detected [52]. Further studies carried out with a higher cellular resolution will be needed in order to get more insight into the intercellular traffic of N metabolites, and especially of photorespiratory NH_4_^+^, in this model legume.

Glutamate dehydrogenase can catalyze *in vitro* both the NADH-dependent incorporation of ammonium into α-KG to give glutamate (aminating reaction) and the NAD-dependent deamination of glutamate to give α-KG and ammonium (deaminating reaction). However, recent evidences indicate that the enzyme works *in vivo* in the direction of glutamate deamination in order to provide α-KG to the Krebs cycle and to control glutamate homeostasis [53, 54]. It may be possible that an increased deamination of glutamate by NAD-GDH activity is needed in order to provide carbon skeletons for the reassimilation of photorespiratory NH_4_^+^ in the mutant. The metabolite profiles presented in this work for the *Ljgln2-2* mutant support this hypothesis, since glutamate levels drop after the shift to active photorespiratory conditions at the same time that α-KG levels are increased. Moreover, the induction of the *LjICDH1* gene also suggested that a cytosolic ICDH isoform may also contribute to the production of α-KG for the reassimilation of NH_4_^+^ in this genotype. Despite of the central role of α-KG in both carbon and nitrogen metabolism, it is still unclear where is the major site of production of this metabolite [55]. Several reports suggested that cytosolic NADP-dependent isocitrate dehydrogenase may synthesize the α-KG necessary for the GS/GOGAT cycles ([42] and references therein). However, no conclusive evidence supporting this idea has been presented up to the date. The induction of *LjICDH1* described here, together with the similar trends of *LjICDH1* transcript levels and α-KG metabolite levels support the idea that cytosolic ICDH may at least in part provide carbon skeletons for NH_4_^+^ re-assimilation in the mutant.

A gene sequence encoding for a putative NADP(H)-GDH was detected in the *L*. *japonicus* genome. When *Ljgln2-2* mutant plants were transferred to active photorespiratory conditions, an induction of NADPH-dependent GDH aminating activity was observed. In addition, a band of the expected size (about 71 kDa) corresponding to NADP(H)-GDH was also detected in the immunoblots. This is, to our knowledge, the first ever identification of the NADP(H)-GDH protein by Western Blot in higher plants. In contrast, when the NADP-dependent deaminating activity was assayed, no detectable enzyme activity was found. These results on the induction of NADP(H)-GDH gene expression, enzyme activity and polypeptide levels in *L*. *japonicus* are very interesting since there is no clear evidence for the presence of an active NADP(H)-dependent GDH in other plant species since very low levels of this enzyme have been found [56]. This may explain also the lack of other reports of any possible cross-reactivity among NAD(H)-GDH antibodies and NADP(H)-GDH. In fact, previous work carried out with *Arabidopsis* could only detect very low levels of NADP-dependent enzyme activity by in-gel GDH activity staining of root, leaves and stem extracts [56]. However, the activity bands observed coincided with these of NAD-GDH ones, suggesting that NADP(H)-dependent GDH is not expressed as an active protein in this plant [56]. The data shown in the present work strongly suggest that NADPH-GDH enzyme is active in *L*. *japonicus* and that this activity is induced in parallel with the lowering of photorespiratory NH_4_^+^ accumulation in the *Ljgln2-2* mutant. Considering that only the aminating and not the deaminating enzyme activity of chloroplastic NADP(H)-GDH was detected, a role of this particular isoenzyme in ammonium reassimilation and/or detoxification in *L*. *japonicus Ljgln2-2* mutants can not be excluded. It could be possible that NADPH-GDH might have an auxiliary role in ammonium detoxification and/or glutamate homeostasis in *L*. *japonicus* chloroplasts, at least when plastidic GS_2_ is absent in this organelle. Further work will be still required to determine the precise role of NADP(H)-GDH in this plant.

Of the three genes encoding for ASN found in the *L*. *japonicus* genome, *LjAsn2* was highly induced in the mutant after the transfer to normal CO_2_ conditions. Considering that the trend in asparagine over time was very similar to the trend in expression level of *LjAsn2* in both genotypes (Fig 4, Table 1), these data suggest that *LjAsn2* may be responsible for the increase in asparagine levels observed in *L*. *japonicus* after the transfer to active photorespiratory conditions. This is consistent with the induction of *LjAsn2* exclusively in the mutant and suggests a role for the corresponding enzyme in supporting NH_4_^+^ detoxification by using part of the Gln formed by the action of GS_1_ in this genotype.

The results shown in this paper indicate that GS_1_/GDH/ASN may have a physiological role in the mutant genotype with the aim of reducing the excessive levels of ammonium. However, in WT plants the normal levels of plastidic GS_2_ seem to be sufficient to efficiently reassimilate the photorespiratory ammonium. Further work would be still required on the diurnal profiling of GS_1_/GDH/ASN that would probably help to get insights into the question whether these genes do show a certain kind of short-term or intermitted response in the WT and therefore would strengthen the physiological role/sense of these alternative enzymes.

Finally, carbamoylphosphate synthase provides carbamoylphosphate for both pyrimidine synthesis pathway [46] and for arginine biosynthesis [57]. Recent studies have shown that CPS can also play a role in NH_4_^+^ detoxification by supporting Arg production [20]. In fact, the CP produced by CPS enzyme activity can be transformed to citrulline that is a precursor of Arg [20]. Of the two genes encoding for CPS in *L*. *japonicus*, only *LjCarB*, which encodes for the large CPS subunit, was significantly modulated by the transfer to active photorespiratory conditions, with a sudden drop in transcript levels followed by a subsequent recovery in both genotypes. CP levels were increased about two times exclusively in the WT genotype by the transfer to active photorespiratory conditions, and were always higher in this genotype compared to the mutant (Table 1). However, no significant differences in gene expression levels were detected among WT and *Ljgln2-2*. Since it was not possible to measure CPS enzyme activity in *L*. *japonicus* leaf extracts, further evidence is needed to support the possible role of CPS in ammonium assimilation and/or detoxification in *L*. *japonicus* plants.

In this work we have analyzed the different enzyme activities and metabolic pathways that may be involved in the reassimilation and detoxification of the photorespiratory NH_4_^+^ that is accumulated in the *Ljgln2-2* mutant. The increase observed for various enzymes and amino acids exclusively in *Ljgln2-2* mutants suggests that nitrogen metabolism is not a limiting factor for the fitness of the mutant and, simultaneously, may constitute a way for ammonium detoxification. Our interpretation of the different observations described in this paper could be summarized as follows: in the absence of plastidic GS_2_ activity, Gln can not be synthesized in the mutant chloroplasts. This may contribute to the increase in α-KG that takes place during the first three days of the transfer from high CO_2_ to normal CO_2_ conditions (Table 1), because of the lack of a functional GS_2_/GOGAT cycle. This may led to an increased cytosolic Gln synthesis by means of cytosolic GS_1_ induction once this Gln is started to be synthesized in the cytosol. Gln should be then redirected to the chloroplast for the purpose of Glu biosynthesis by means of a combined GS_1_/GOGAT cycle. This may explain the gradual drop in α-KG levels observed after day 3 (Table 1). This should contribute to Glu homeostasis that is fundamental for the plant since Glu is the precursor of several amino acids [58]. The increased cytosolic Gln produced from photorespiratory NH_4_^+^ may also support Asn biosynthesis by the action of ASN. This is important since Asn plays a fundamental role in nitrogen transport and remobilization in *L*. *japonicus* [59], and may also contribute to the drop in Glu levels observed in the mutant, since Asp, one of the substrates of ASN, is synthesized from oxaloacetate and Glu. The induction of genes encoding for different NAD(H)-dependent GDH isoforms and the increase of the corresponding polypeptide levels and enzyme activity is very probably aimed to provide carbon skeletons for photorespiratory NH_4_^+^ reassimilation and, on the other hand, to fuel the Krebs cycle under conditions in which many photosynthetic genes are repressed [18]. This catabolic role of GDH would be in accordance with the most recent results proposed for this enzyme in plants [53, 54] and would account also for the drop in Glu observed in the *Ljgln2-2* mutant plants. A canalization of the C contained in the amino acids to the Krebs cycle and finally a higher production of transport and reserve amino acids such as Gln and Asn would be expected. Our data also suggested that a cytosolic ICDH isoform may contribute to the production of carbon skeletons for the re-assimilation of NH_4_^+^. On the other hand, another interesting feature of the present paper is the finding of an active chloroplastic NADP(H)-GDH which may have an auxiliary role in ammonium detoxification and/or glutamate biosynthesis/homeostasis in *L*. *japonicus* chloroplasts. The results presented in this work make a significant improvement in the study of nitrogen reassimilation in *L*. *japonicus* and of the regulation of the key enzymes of this process.

## Conclusions

An increase in the corresponding enzyme activity and/or transcription was found for several genes encoding for enzymes that may constitute an alternative way for photorespiratory ammonium reassimilation and detoxification in *L*. *japonicus* plants in the absence of plastidic GS_2_. The induction of cytosolic GS_1_ suggests that, this key enzyme of N metabolism may also be involved in the reassimilation of photorespiratory ammonium when plastidic GS_2_ is lacking and may be responsible for the increase in the levels of glutamine observed in the mutant plants. GDH enzyme activity and ASN gene expression were also higher in the mutant, indicating that these enzymes may act in conjunction with GS_1_ to cope with the process of photorespiratory ammonium accumulation in the plants. In addition, the increase in the polypeptide levels and aminating enzyme activity of NADP(H)-GDH discovered in the *Ljgln2-2* mutant plants under photorespiratory active conditions, constitutes a very novel and important finding of the present work.

## Supporting Information

S1 Fig Experimental design used in this work. WT and mutant plants were grown under high CO2 conditions (0.7% v/v) for 35 days and then transferred to normal CO2 conditions (0.04% v/v). Leaf samples from three different biological replicates were taken at time 0 (high CO2) and at the time points in days that are indicated under normal CO2.
(PPT)

S2 Fig Multiple sequence alignment of the four genes encoding for GDH in *L*. *japonicus*. The alignment was carried out using ClustalW with the Gonnet protein weight matrix and a gap open penalty of 10. The theoretical molecular weights of the polypeptides are of 44.74, 44.72, 44.66 and 70.74 kDa for *LjGDH1*, *LjGDH2*, *LjGDH3* and *LjGDH4* respectively. A dehydrogenase dimerization domain (pfam02812) between the amino acids 240 and 360 of *LjGDH4* and the NAD(P)-binding domain of GDH from the amino acid 379 to the end of *LjGDH4* were detected using the BLASTP program at NCBI (http://blast.ncbi.nlm.nih.gov/Blast.cgi).
(PPT)

S1 Table Oligonucleotides used for qRT-PCR. (DOC)
