# Correction: Reassimilation of Photorespiratory Ammonium in *Lotus japonicus* Plants Deficient in Plastidic Glutamine Synthetase

**DOI:** 10.1371/journal.pone.0156568

**Published:** 2016-05-27

**Authors:** Carmen M. Pérez-Delgado, Margarita García-Calderón, Antonio J. Márquez, Marco Betti

There are errors in the Funding section. The correct funding information is as follows:

This work was supported by project P1O-CVI-6368 and BIO-163 support from Consejería de Economía, Innovación y Ciencia, Junta de Andalucía (http://www.juntadeandalucia.es/organismos/economiainnovacioncienciayempleo.html) and by the project AGL2014-54413-R from FEDER-Ministerio de Economía y Competitividad (http://www.mineco.gob.es/). CMP acknowledges the support of PIF and V Plan Propio fellowships from the University of Seville (www.us.es [1]). The funders had no role in study design, data collection and analysis, decision to publish, or preparation of the manuscript.
