# Supplementary material for: Reassimilation of Photorespiratory Ammonium in Lotus japonicus Plants Deficient in Plastidic Glutamine Synthetase
Source: PLoS One. 2015 Jun 19;10(6):e0130438. doi: 10.1371/journal.pone.0130438 (PMC4474828; doi:10.1371/journal.pone.0130438)
Supplement: S1 Fig — WT and mutant plants were grown under high CO2 conditions (0.7% v/v) for 35 days and then transferred to normal CO2 conditions (0.04% v/v). Leaf samples from three different biological replicates were taken at time 0 (high CO2) and at the time points in days that are indicated under normal CO2. (PPT) [file pone.0130438.s001.ppt]

## Slide 1
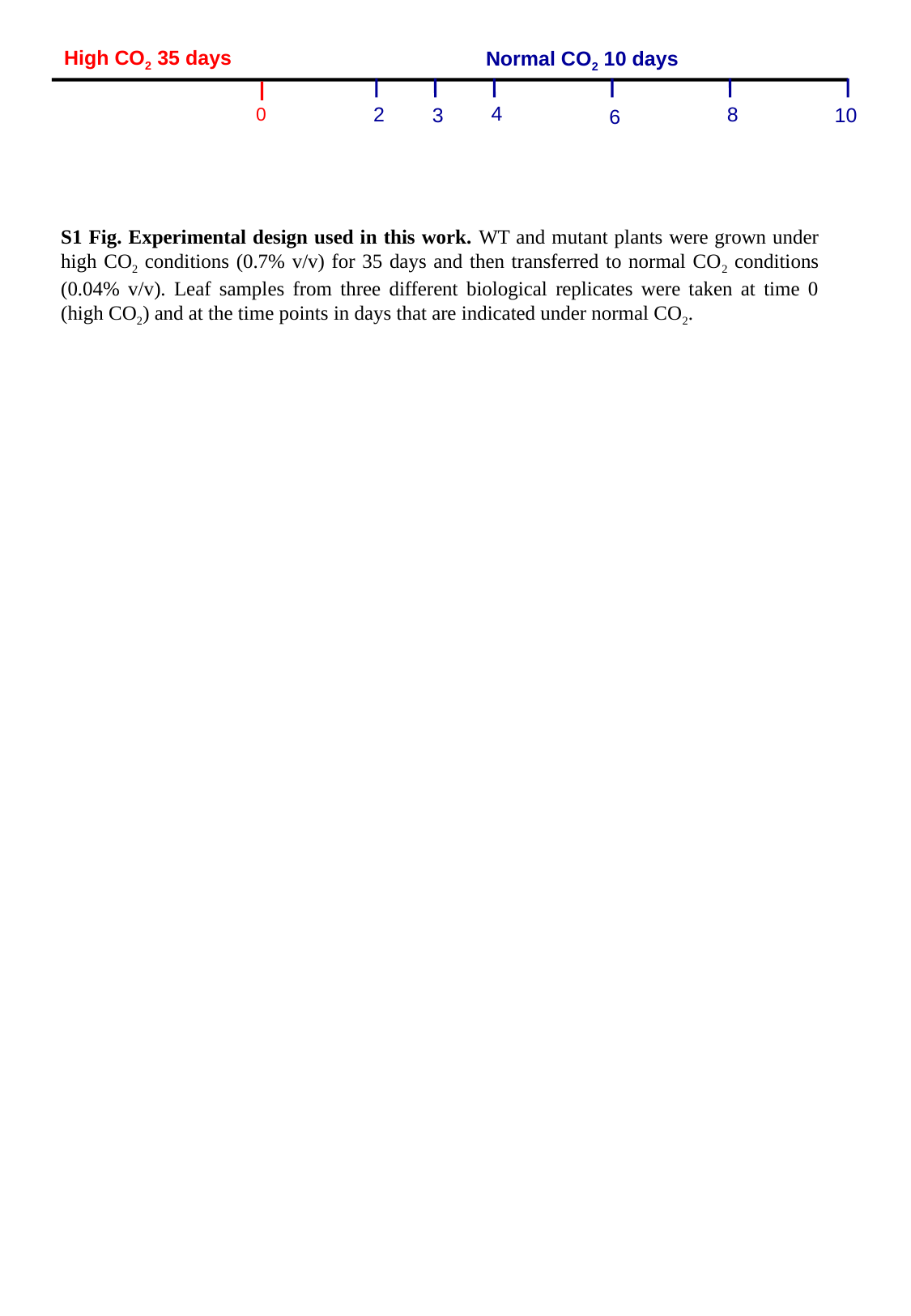

High CO2 35 days
Normal CO2 10 days
4
2
8
0
3
10
6
S1 Fig. Experimental design used in this work. WT and mutant plants were grown under high CO2 conditions (0.7% v/v) for 35 days and then transferred to normal CO2 conditions (0.04% v/v). Leaf samples from three different biological replicates were taken at time 0 (high CO2) and at the time points in days that are indicated under normal CO2.
