# Supplementary material for: Reassimilation of Photorespiratory Ammonium in Lotus japonicus Plants Deficient in Plastidic Glutamine Synthetase
Source: PLoS One. 2015 Jun 19;10(6):e0130438. doi: 10.1371/journal.pone.0130438 (PMC4474828; doi:10.1371/journal.pone.0130438)
Supplement: S2 Fig — The alignment was carried out using ClustalW with the Gonnet protein weight matrix and a gap open penalty of 10. The theoretical molecular weights of the polypeptides are of 44.74, 44.72, 44.66 and 70.74 kDa for LjGDH1, LjGDH2, LjGDH3 and LjGDH4 respectively. A dehydrogenase dimerization domain (pfam02812) between the amino acids 240 and 360 of LjGDH4 and the NAD(P)-binding domain of GDH from the amino acid 379 to the end of LjGDH4 were detected using the BLASTP program at NCBI (http://blast.ncbi.nlm.nih.gov/Blast.cgi). (PPT) [file pone.0130438.s002.ppt]

## Slide 1
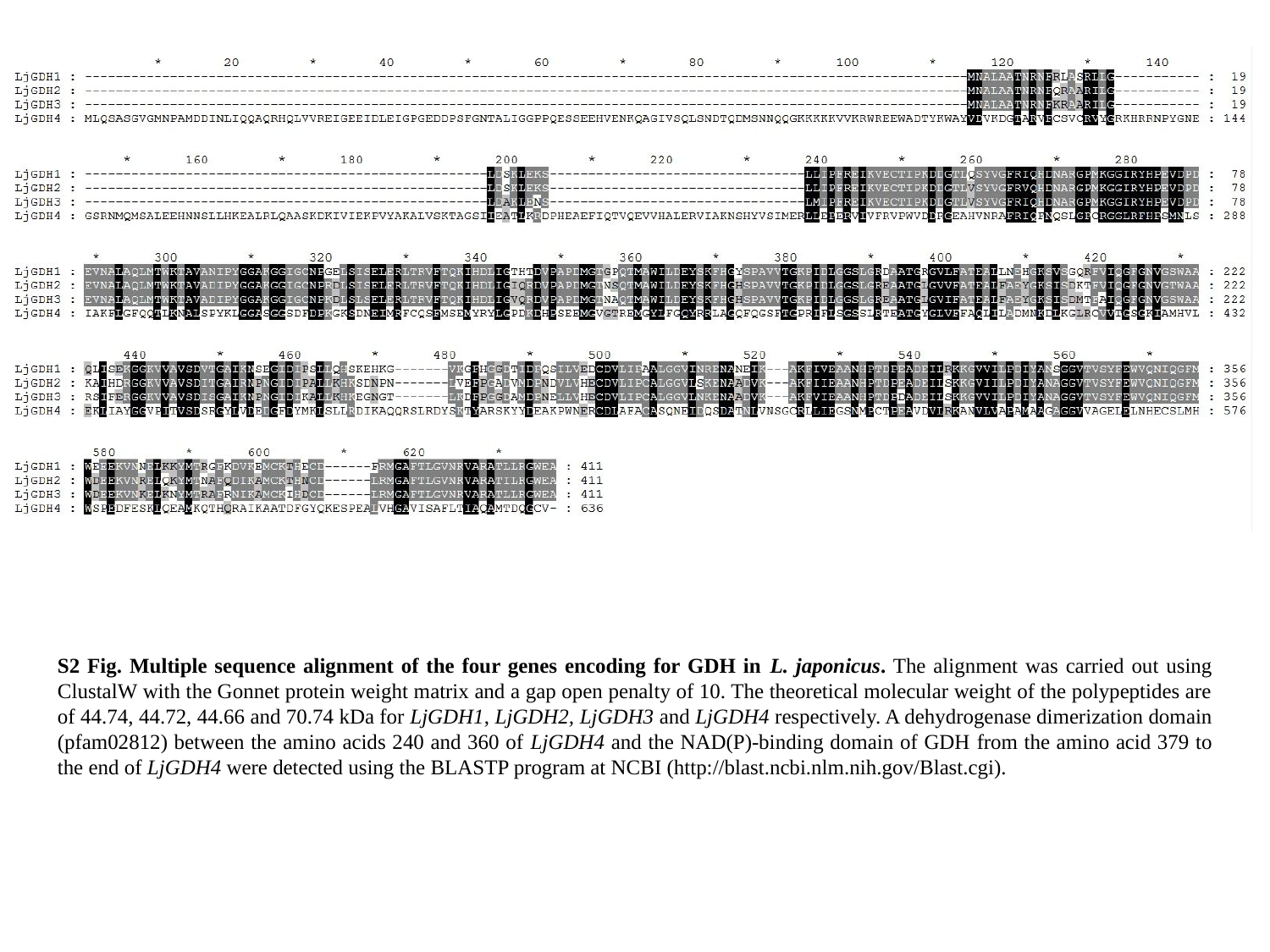

S2 Fig. Multiple sequence alignment of the four genes encoding for GDH in L. japonicus. The alignment was carried out using ClustalW with the Gonnet protein weight matrix and a gap open penalty of 10. The theoretical molecular weight of the polypeptides are of 44.74, 44.72, 44.66 and 70.74 kDa for LjGDH1, LjGDH2, LjGDH3 and LjGDH4 respectively. A dehydrogenase dimerization domain (pfam02812) between the amino acids 240 and 360 of LjGDH4 and the NAD(P)-binding domain of GDH from the amino acid 379 to the end of LjGDH4 were detected using the BLASTP program at NCBI (http://blast.ncbi.nlm.nih.gov/Blast.cgi).
