# Supplementary material for: Reassimilation of Photorespiratory Ammonium in Lotus japonicus Plants Deficient in Plastidic Glutamine Synthetase
Source: PLoS One. 2015 Jun 19;10(6):e0130438. doi: 10.1371/journal.pone.0130438 (PMC4474828; doi:10.1371/journal.pone.0130438)
Supplement: S1 Table — (DOC) [file pone.0130438.s003.doc]

| **Gene** | **Kazusa 2.5 code** | **Primers** | |
| --- | --- | --- | --- |
|  |  | **Forward** | **Reverse** |
| ***LjGln1.1*** | *chr2.CM0312.1480.r2.m* | TGGACCACAGGGCCCATAC | AATGTCACGCCCATAGGCTTT |
| ***LjGln1.2*** | *chr6.CM0014.300.r2.m* | TGAGGTGTGGGTTGCTCGTT | AAGGACCACCCCAGCAATCT |
| ***LjGln1.3*** | *LjSGA_030247.1* | TAACCTCTCCGAGACCACCG | CCAATCCATATGTATTCGGCG |
| ***LjGln1.4*** | *LjSGA_058827.1* | AGAGAGACTGAGAAAGATGGAAAATGT | CAGGCCTCCTCTGTCCTCAA |
| ***LjGln1.5*** | *LjSGA_019428.1* | GGGTAGGCAGGGAGACTGAAA | GAAGCTGGCCTCCTGTCCTC |
| ***LjGdh1*** | *chr1.CM0104.2530.r2.m* | GGAGATGTGCAAAACCCATGA | CGGTTAACTCCCAGGGTGAAA |
| ***LjGdh2*** | *chr4.CM2142.210.r2.a* | ACATGACAATGCTCGTGGTCC | AGGGTCAACCTCAGGGTGGTA |
| ***LjGdh3*** | *chr2.CM0021.1320.r2.m* | GCTGCTGATGTGAAAGCGAAA | GCATCTGGGTCAGTGGGATG |
| ***LjGdh4*** | *chr3.CM1488.210.r2.d*  *chr3.CM1488.260.r2.d*  *chr3.CM1488.250.r2.d*  *chr3.CM1488.230.r2.d* | AAGGTTCAAACATGCCCTGCAC | AGCAGGAGCAACGAGAACATTAGC |
| ***LjAsn1*** | *chr5.CM0071.330.r2.d* | TGGAGGACCAACTGTTGCATG | AGACCAAGCAGCATCCCACTC |
| ***LjAsn2*** | *LjT47C13.80.r2.d* | TCAGTGAGCAAAGGTGTTGAACC | CAAGAGGAGAAACTTCCATCTTGG |
| ***LjAsn3*** | *LjT09J04.190.r2.d* | CGAACTGGCAGTGATTGTGAAGTG | ATGCCAATAGCATCACGAGCAG |
| ***LjCarA*** | *chr1.LjT17C10.30.r2.d* | TGGGAATGGAGATAGGCCTTGG | TGAACCATCCTCCAGCACAAGC |
| ***LjCarB*** | *LjSGA_053625.1* | GGGACTACCCTTGAGGAGTGTTTG | TAAACGCGGGCCTAACGATCAC |
| ***LjICDH1*** | *chr1.CM0023.360.r2.m* | AAGCAGCCCATGGCACAGTTAC | ATGCTGTTTGTGCTGGTTTCCC |
| ***LjIDH1*** | *chr5.CM1729.170.r2.m* | AGATCGCCGAATCCGTCAAACAG | TCGGTCCCTACATAGTGCTCTTCC |
| ***LjIDH2*** | *chr2.CM0272.230.r2.d* | ACTGCATCAACAACCTCTTGCG | CAAGTCATTCTAGAGGGCAAGCAC |

**S1 Table. Oligonucleotides used for qRT-PCR.**
